# Improving phosphorus efficiency in cereal crops: Is breeding for reduced grain phosphorus concentration part of the solution?

**DOI:** 10.3389/fpls.2013.00444

**Published:** 2013-11-05

**Authors:** Terry J. Rose, Lei Liu, Matthias Wissuwa

**Affiliations:** ^1^Southern Cross Plant Science, Southern Cross UniversityLismore, NSW, Australia; ^2^Southern Cross GeoScience, Southern Cross UniversityLismore, NSW, Australia; ^3^Stable Food Production Program, Japan International Research Center for Agricultural SciencesIbaraki, Japan

**Keywords:** grain phosphorus, nutrient use efficiency, phosphorus cycle, phosphorus utilization efficiency, sustainable agriculture

## Abstract

Given the non-renewable nature of global phosphate reserves, there is a push to increase the phosphorus (P) efficiency of agricultural crops. Research has typically focussed on investigating P acquisition efficiency or internal P utilization efficiency to reduce crop fertilizer requirements. A novel option that would reduce the amount of P exported from fields at harvest, and may ultimately reduce P fertilizer requirements, would be to reduce the amount of P translocated to grains to minimize grain P concentrations. While such a trait has been mentioned in a number of studies over the years, there has not been a concerted effort to target this trait in breeding programs. In this perspective piece we explore the reasons why a low grain P trait has not been pursued, and discuss the potential benefits and drawbacks of such a trait in the context of breeding to improve the P efficiency of cropping systems.

## INTRODUCTION

The majority of the world’s mined rock phosphate is used for the manufacture of phosphorus (P) fertilizers applied in agriculture to improve or sustain crop yields. Over 17.5 mt P per year was used for agricultural purposes from 2004 to 2008, and this figure is projected to increase over the coming years (Lott et al., 2011). While P fertilizer prices remained at a stable and low level for several decades, they have more than doubled recently and considering the non-renewable nature of rock-P resources, further price increases seem inevitable (Cordell et al., 2009). Increasing the efficiency with which P is used in agricultural systems is therefore critical for sustainable food and fiber production in the twenty-first century.

Crop P efficiency (PE) can improve if either yields increase at a given rate of P fertilizer application, or if yields remain stable with lower levels of P fertilizer application. This could be broadly achieved by enhancing P uptake (P acquisition efficiency; PAE) or by improving internal P utilization efficiency (PUE). Given that only a portion of the P applied in fertilizers is actually taken up by a crop on high P fixing soils, with the remaining P being slowly immobilized in the soil, there seems to be opportunity for further improvement in PAE. Several reviews on the topic of enhancing PAE have recently been published and the reader is referred to Richardson et al. (2009) for further discussion.

Far less research has been conducted on the topic of how to improve PUE. While numerous agronomic definitions of PUE exist (e.g., grain yield per unit of P fertilizer applied, grain yield per unit of P in aboveground biomass), from a physiological and breeding perspective we define PUE as shoot biomass produced per unit P in shoots (Rose and Wissuwa, 2012). Essentially, plants with higher PUE operate at lower shoot P concentrations. During the vegetative growth phase most plant P is contained in shoot tissue and shoot P concentrations (as opposed to root P concentrations) are therefore of primary importance for improved PUE (Rose and Wissuwa, 2012). However, at harvest, cereals typically contain 70^+^% of their total P in grains with very little remaining in straw, prompting us to investigate whether reductions in grain P concentrations are a possible way to improve overall crop PE in cereal systems. Previous studies have already demonstrated that grain P can be lowered through recurrent selection (Wardyn and Russell, 2004) or by mutation (Raboy, 2009). In this opinion piece, we discuss the advantages and possible disadvantages of a low grain P trait in the context of breeding P-efficient crops for sustainable cropping systems.

## REDUCING P FLOWS THOUGH CROPPING SYSTEMS: IMPLICATIONS FOR FERTILIZER REQUIREMENTS AND THE ENVIRONMENT

The importance of P in harvested products is highlighted by calculations showing that the total P removed annually in grain and fleshy fruit crops equates to 85% of fertilizer P applied to crops globally (Lott et al., 2009). While this calculation would suggest that the global P balance is positive, it has to be kept in mind that huge imbalances in the application of P fertilizer exist across the globe, with P surpluses in many European and East-Asian countries and large P deficits in many poorer areas including sub-Saharan Africa (MacDonald et al., 2011). The high P removal rate in harvested grains drives the need to replace soil-P by fertilizer application or leads to P mining where fertilizer application rates are low. Developing crop varieties that translocate less P to developing grains may offer one option to balance P budgets in agriculture at reduced fertilizer requirements.

**FIGURE 1:**
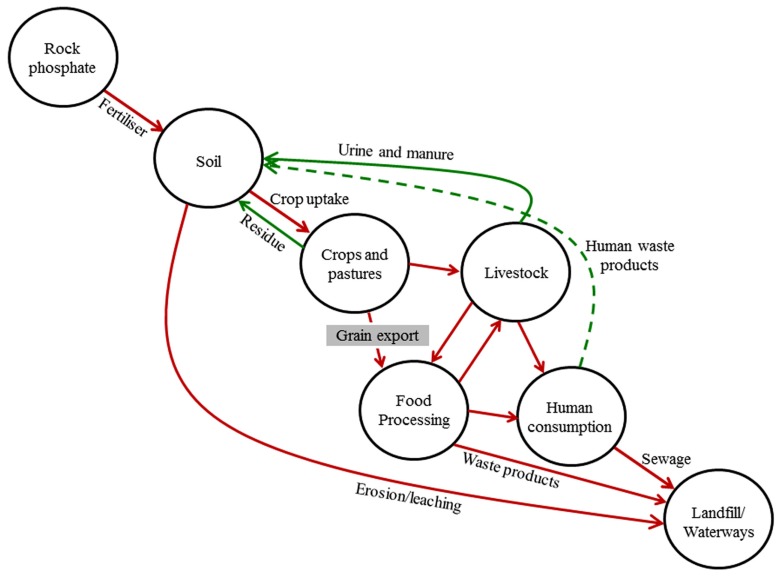
**Major flows of phosphorus in the global agricultural phosphorus cycle.** Broken green line indicates a major break in the cycle due to poor recovery and recycling of human waste; gray box indicates potential point to slow the flow of P through agricultural systems.

Low grain P crop varieties may also have environmental benefits because much of the P removed from fields ultimately ends up in landfill or water bodies via sewage pathways (Cordell et al., 2009). This occurs because there are losses of P through inefficiencies at each step in the food chain, including inefficient recovery or suboptimal redistribution of high-P animal wastes, losses of P during food manufacturing processes, and particularly poor recovery and use of P in human waste (**Figure 1**). One of the major causes of inefficiency is phytate, the organic storage form of P which usually accounts for around 75% of total P in grains, because animals – particularly monogastric animals including humans – cannot properly digest phytate, leading to high P concentrations in feces and urine (Raboy, 2007). In the case of livestock, the poor digestibility of phytate can also lead to P deficiency in the animals, which has traditionally been rectified by using P feed supplements or by the addition of phytase enzymes to feed rations (Raboy, 2009). More recently, low phytic acid (*lpa*) mutants have been identified in major crop species, which typically have a reduced concentration of phytate with concurrent increases in inorganic P in grains, providing better P nutrition in livestock and a reduction of P in feces and urine (Raboy, 2009).

In contrast to livestock, humans rarely suffer from P deficiency (see “Grain P implications for human health” below), so additional inorganic P in grains for human consumption is of little benefit and P concentrations in human waste will remain high. While recycling of P in human and animal waste needs to be improved globally to alleviate environmental effects and close the P loop (**Figure 1**), it seems unlikely that it will occur rapidly and efficiently enough over the coming decades to prevent substantial loss of valuable P from agricultural systems. Thus, attempting to minimize the throughput of P in the agricultural sector would be the best way of minimizing negative environmental impacts while maintaining high productivity. The most logical way to reduce the throughput is to address the removal of P from farms in harvested grain (**Figure 1**).

Interestingly, the concept of reducing grain P has been raised on numerous occasions in the past (e.g., Batten, 1992; Ockenden et al., 2004; Raboy, 2009) but there has not yet been a global effort to address the issue, presumably because of the association between seed P levels and seedling vigor. We argue that given the economic and environmental gains to be made with a low grain P trait, seedling vigor should not be seen as an impediment for further exploration, but rather an issue that should also be addressed through further research.

## PHOSPHORUS EFFECTS ON SEED GERMINATION AND SEEDLING VIGOR

Early crop vigor is critical in productive farming systems and typically relates to higher biomass and/or grain yields. Sustaining or increasing seed P content is thought to improve seedling establishment and vigor (Bolland and Baker, 1988; White and Veneklaas, 2012). Two separate lines of inquiry have contributed to these conclusions: the first of these was a series of studies from the 1980s and 1990s which reported that seeds obtained from plants grown in P-deficient soil (which had lower P concentrations than seeds obtained from plants grown in high-P soils) had poor germination and establishment, and subsequent lower biomass or grain yields (Bolland and Baker, 1988; de Marco, 1990; Burnett et al., 1997; Derrick and Ryan, 1998; Zhu and Smith, 2001).

We recently conducted a study using low-P rice seed obtained from plants grown in P-deficient soil and found that seed germination and seedling vigor were impaired, as per the earlier studies mentioned above (Rose et al., 2012). However, we concluded that a significant proportion of the reduced germination and seedling vigor was likely due to the severe P deficiency stress suffered by the mother plants during grain filling, rather than caused by low seed P concentration *per se*. Further experiments using seeds differing in P concentration that were not obtained from plants grown under severe P deficiency found no differences in yields when plants were grown in P-replete or P-deficient soil (Rose et al., 2012). More recently, experiments have suggested that genotypic differences have a much larger effect on seedling vigor than seed P concentrations or total seed P content. Even seed P concentrations as low as 1 mg g^-^^1^ did not impair germination, seeding vigor, or final grain yield in a generally vigorous rice genotype, presumably because such vigorous genotypes rapidly compensate for low seed P by seedling P uptake (Wissuwa, unpublished data).

A second line of enquiry using *lpa* mutants or transgenic plants with altered phosphatase scavenging capacity has also suggested that alterations to seed P affect seed germination and seedling vigor (Raboy, 2009; Robinson et al., 2012). Studies with *lpa* mutants across a range of species found reduced seedling vigor or impaired germination compared to wild-type parent lines (Meis et al., 2003; Kim and Tai, 2011). However, most *lpa* mutations impair the function of synthase or kinase enzymes involved in inositol phosphate metabolism, and this metabolism is important in vegetative tissues as well as developing seeds. Hence, perturbation of the phytic acid synthesis pathway can have downstream effects on seed viability, germination and seedling vigor, and general plant growth, especially under stress conditions (Raboy, 2009). Similarly, Robinson et al. (2012) recently reported that eliminating the purple acid phosphatase, AtPAP26, in *Arabidopsis thaliana* decreased seed P levels but also reduced seed germination. These authors concluded that reducing P translocation to the seed is undesirable; however, the poor germination could also be explained by the fact that AtPAP26 probably has a critical role in the seed germination process.

In all of the above-mentioned cases, it is plausible that the disruption of key metabolic pathways induced by mutation leads to problems with seed germination and seedling vigor rather than a deficiency of P in seeds *per se*. Indeed, proof-of-concept that a reduction in seed total P does not necessarily impact on subsequent crop performance is provided by the barley *lpa1-1* mutant (Raboy, 2009). This mutant has a 10–20% reduction in seed P, and barley isolines with the mutation do not suffer from an obvious yield penalty across a range of environments (Bregitzer and Raboy, 2006). Importantly, the mutation – to a gene which appears to encode for a member of the sulfate transporter gene family (Ye et al., 2011) – appears to have a seed-specific impact by reducing endosperm P levels by 30% (Ockenden et al., 2004) without broader negative effects on plant physiological processes. The fact that commercial barley cultivars have been developed and released in North America (Bregitzer et al., 2007) suggests that reducing the P concentration of cereal crops by around 20% through breeding without reducing subsequent crop yields is certainly feasible.

## THE NEED FOR A LOW GRAIN P TRAIT TO IMPROVE INTERNAL PUE

Internal PUE has received significant attention recently due to recognition that enhanced PAE alone cannot improve the PE of farming systems (Rose et al., 2011; Veneklaas et al., 2012). However, the target of improving PUE, i.e., increasing biomass production per unit P, may clash with the perceived need to maintain high grain P concentrations. This issue is rather elegantly explained by Barraclough et al. (2010) with regard to internal nitrogen use efficiency (NUE) and grain protein concentrations, whereby high NUE is likely to come at the cost of reducing grain protein (Barraclough et al., 2010). These authors applied what is known as the “law of conservation of matter” to NUE, and the concept applies to any nutrient, including P. Reduced shoot P concentrations (higher PUE) will logically lead to reduced grain P concentrations unless some way can be found to uncouple both traits. Such uncoupling could theoretically be achieved by increasing the proportion of P located in grains at harvest (P Harvest Index; PHI). However, given that the PHI of most crops is already general above 70% and can be up to 90% (Batten, 1992; Rose et al., 2007,^2008^; Bi et al., 2012), scope to enhance this further appears rather limited.

We have simulated PUE improvements in rice permissible under the “law of conservation of matter” based on different grain P and PHI scenarios (**Table 1**). Calculations suggest that if grain P concentrations were sustained at a given level (e.g., 2.5 mg g^-^^1^), an increase in PHI from 0.7 to 0.8 would allow for moderate reductions in shoot P concentration at flowering – i.e., an increase in vegetative PUE (**Table 1**). However, much larger gains in PUE at flowering are only possible if grain P concentrations are reduced (**Table 1**). Rose et al. (2010) suggested a breeding target of a 20% reduction in grain P in rice (e.g., from 2.5 to 2.0 mg g^-^^1^), which, using the rough calculations in **Table 1**, would allow for a reduction in shoot P concentration at flowering of between 0.4 and 0.7 mg g^-^^1^, depending on the PHI and amount of P taken up from the soil during grain filling. While shoot P concentrations of most cereal crops range from around 4 mg g^-^^1^ at tillering to around 2 mg g^-^^1^ at flowering, as little as 1 mg g^-^^1^ P may actually be necessary for normal cellular function (Veneklaas et al., 2012). This is consistent with reports that reduced growth under P deficiency may be due to gene expression reprogramming rather than a direct result of low levels of shoot P (Rouached et al., 2011). Given the fact that it may be possible to repress the gene expression reprogramming observed under P deficiency (Rouached et al., 2011) it appears that there is a real prospect of improving PUE in crop plants. However, any reduction in shoot P concentrations toward the theoretical limit of around 1 mg g^-^^1^ will not be possible without a subsequent reduction in grain P concentration.

**Table 1:** Simulated allowable changes in shoot P concentration at flowering with varying grain P concentrations or phosphorus harvest index (PHI).

Grain yield (kg ha^–1^)	Grain P concentration (mg g^–1^)	Grain P content (kg P ha^–1^)	Total crop P uptake (kg P ha^–1^)		Shoot P concentration at flowering (mg g^–1^)^a^		Shoot P concentration at flowering (mg g^–1^)^b^	
			PHI 0.7	PHI 0.8	PHI 0.7	PHI 0.8	PHI 0.7	PHI 0.8
7000	2.5	17.5	25.0	21.9	3.6	3.1	2.4	2.1
	2	14.0	20.0	17.5	2.9	2.5	1.9	1.7
	1.5	10.5	15.0	13.1	2.1	1.9	1.5	1.3

*The simulation presumes plants have a harvest index of 0.5, and that the PHI is not affected directly by grain yield or total P uptake. Shoot P concentrations are calculated as total crop P uptake at flowering / straw biomass at flowering.*

a Assumes straw biomass does not change from flowering to harvest and no additional P uptake after flowering.

b Assumes straw biomass 10% higher at flowering compared to harvest and 25% of P uptake after flowering.

## P EFFICIENCY VERSUS P BALANCE EFFECTS

Grain P concentrations could therefore, in theory, be reduced passively as the result of improvements to crop PUE or actively by breeding specifically for a low grain P trait. If a reduction in grain P concentrations is the result of improvements to PUE, one could expect that there would be a decline in P fertilizer requirements (because presumably the high PUE plants would not need to acquire as much soil P as current cultivars), as well as a “slowdown” in global P cycling as the throughput of P is minimized. The reduced throughput of P in the system would be driven by the lower P fertilizer requirements of crops but the low grain P trait would also minimize the subsequent P flow through the food chain. However, in reality breeding for improvements to PUE may be challenging in high-input farming systems (Rose and Wissuwa, 2012). In the absence of enhanced PUE, breeding directly for a low grain P trait would still reduce P flow through the food chain and minimize soil P mining, but whether the return of high-P straw residue to the soil could reduce subsequent P fertilizer requirements is not known.

One question that must be addressed is whether additional P retained in straw would be bioavailable or whether it would simply contribute to the build-up of recalcitrant organic P in soils. Given that contamination of waterways with P from diffuse sources (e.g., agricultural runoff) is the major contributor to eutrophication (Carpenter et al., 1998), the continued build-up of P in soils is a major concern. Recalcitrant P in soils is a particular problem because it is of little benefit to plants, but can become biologically active while residing in the sediment layer at the bottom of water bodies (Correll, 1998). However, the general perception that the bulk of P returned to soil in organic material is in an organic form is largely unfounded (McLaughlin et al., 2011) and much of it is present as inorganic P or readily degradable organic P forms (Noack et al., 2012). Although inorganic P from crop residues can become immobilized in soil organic P through microbial processes, the same processes also apply to P applied as inorganic or organic fertilizer. Ultimately, the value of P returned in crop residues may depend on breeding crops that can utilize these P sources directly or indirectly through microbial mediated pathways (Richardson et al., 2011).

## GRAIN P IMPLICATIONS FOR HUMAN HEALTH

Phosphorus is the second most abundant mineral in the human body after calcium, and 85% of it exists in bone. Dietary P intake is critical because both P deficiency and excess can damage bone health (Takeda et al., 2012; Calvo and Uribarri, 2013). The average recommended P intake is 700 mg/day, varying from 100 mg/day infants to 1250 mg/day for 9 to 18-year-olds (Willett and Buzzard, 1998). The greatest contributors to P intake are protein-rich foods (e.g., dairy products) and cereal grains (Takeda et al., 2002; Welch et al., 2009). The P intake from cereals alone can reach 400–600 mg/day in some Asian countries such as Japan where cereals (e.g., rice) are a staple food (Takeda et al., 2002). Therefore, reducing P concentrations or modifying the composition of P-containing compounds in grains may have significant effects on human health which warrants careful consideration.

Phosphorus in cereal grains is present in different chemical forms including inorganic phosphate, phytate, phospholipids, DNA, RNA, and ATP (Raboy, 2009), though phytate and phospholipids have the most impact on human health (Kumar et al., 2010; Liu et al., 2013). While phytate is poorly digestible and regarded as an anti-nutrient because it reduces the bioavailablity of micronutrients and protein, it has been reported to have positive effects – it may protect against cancer and may help treat diabetes mellitus, atherosclerosis and heart disease (Kumar et al., 2010). However, these authors note that the effective dosage to elicit these beneficial effects is still not clear. Compared to phytate, cereal phospholipids provide a better source of bioavailable P and vitamins such as choline. Dietary phospholipids have beneficial effects on many human diseases including coronary heart disease, cancers, and inflammation (Kullenberg et al., 2012). Cereal starch also contains a significant portion of lysophospholipids which naturally form an inclusion complex with amylose (Liu et al., 2013). When the lysophospholipids form a complex with amylose, they may slow down the rate of digestion and adsorption of starch which may be beneficial to diabetics (Holm et al., 1983).

While there has been a substantial effort to breed or engineer crops with reduced phytate but unchanged total P in grains (Raboy, 2009), the impact of such crops on human health are still unknown. As phospholipids have clearer nutritional values and health benefits than either phyate or inorganic phosphorus, there may be scope to breed healthier cereal grains by lowering phytate but increasing phospholipids contents, in particular the starch lysophospholipids which are located in the endosperm (i.e., in white rice, which is most commonly consumed). Research into grain phospholipids and the potential to maintain or increase the concentration of starch phospholipids while reducing phytate is ongoing.

## CONCLUSION

Phosphorus is a non-renewable resource and recycling is unlikely to turn the inefficient “open ended P cycle” into a closed “cycle” in the near future. A low grain P trait derived indirectly by improving PUE in crops, or directly through targeted breeding for low grain P concentrations, may reduce the throughput of P in agricultural systems. A number of issues are yet to be resolved. First and foremost, the relationship between seed P concentrations and seedling vigor needs to be clarified in a series of environments and genotypes. Should negative associations be detected, in some cases they may be overcome through research on seedling nursery management and seed treatments like coating or priming. The second main area of research would address the question of whether natural genetic variation exists for a low-P trait within crops that could be exploited by breeders immediately. Alternatively, one may focus on creating novel variation through mutations or other genetic modifications, or explore whether grain P concentrations are similar to other grain traits like protein content that can be altered over several cycles of recurrent selection. We believe the financial and environmental gains to be made by introducing a low grain P trait are sufficiently high to warrant pursuing this trait through further research.

## Conflict of Interest Statement

The authors declare that the research was conducted in the absence of any commercial or financial relationships that could be construed as a potential conflict of interest.
